# Tissue-Resident Memory-Like CD8^+^ T Cells Exhibit Heterogeneous Characteristics in Tuberculous Pleural Effusion

**DOI:** 10.1155/2021/6643808

**Published:** 2021-04-22

**Authors:** Sifei Yu, Suihua Lao, Binyan Yang, Changyou Wu

**Affiliations:** ^1^Institute of Immunology, Zhongshan School of Medicine, Sun Yat-sen University, 74 Zhongshan 2nd Road, Guangzhou 510080, China; ^2^Clinical Research Institute, The First People's Hospital of Foshan, 81 Lingnan Road, Foshan 528000, China; ^3^Chest Hospital of Guangzhou, 62 Hengzhigang Road, Guangzhou 510095, China; ^4^Clifford Hospital, Jinan University, No. 3 Hongfu Road, Panyu, Guangzhou 511495, China

## Abstract

Tissue-resident memory T (T_RM_) cells are well known to play critical roles in peripheral tissues during virus infection and tumor immunology. Our previous studies indicated that CD69^+^CD4^+^ and CD69^+^CD8^+^ T cells in tuberculous pleural effusion (TPE) were antigen-specific memory T cells. However, the phenotypical and functional characteristics of CD8^+^ T_RM_ cells in tuberculosis remain unknown. We found that CD103^+^CD8^+^ T cells were the predominant subset of CD103^+^ lymphocytes in TPE; both CD103 and CD69 expressed on memory CD8^+^ T cells from TPE were significantly increased compared with those from paired peripheral blood. Phenotypically, CD103^+^CD69^+^ and CD103^+^CD69^−^CD8^+^ T cells expressed higher levels of CD45RO than CD103^−^CD69^+^CD8^+^ T cells did; CD103^+^CD69^−^CD8^+^ T cells highly expressed CD27, CD127, and CD62L and some chemokine receptors. We further compared the functional differences among the four distinct CD45RO^+^CD8^+^ T subsets identified by CD103 and CD69 expression. In consist with our published results, CD69^+^CD8^+^ T cells, but not CD103^+^CD8^+^, produced high levels of IFN-*γ* after treatment with BCG in the presence of BFA. Nevertheless, CD103^−^CD69^+^ and CD103^+^CD69^+^ memory CD8^+^ T cells expressed higher levels of Granzyme B, while CD103^+^CD69^−^ memory CD8^+^ T cells were characterized as a possibly immunosuppressive subset by highly expressing CTLA-4, CD25, and FoxP3. Furthermore, TGF-*β* extremely increased CD103 expression but not CD69 *in vitro*. Together, CD103^+^CD8^+^ T cells form the predominant subset of CD103^+^ lymphocytes in TPE; CD103 and CD69 expression defines distinct CD8^+^ T_RM_-like subsets exhibiting phenotypical and functional heterogeneity. Our findings provide an important theoretical basis to optimize and evaluate new tuberculosis vaccines.

## 1. Introduction

Tuberculosis (TB) is a communicable disease caused by the *Mycobacterium tuberculosis* (*M.tb*), an estimated 10.0 million (range, 8.9~11.0 million) people fell ill with TB in 2019, and the number has been declining very slowly in recent years [1, 2]. Primary tuberculous pleural effusion (TPE) occurs as a result of a delayed hypersensitivity reaction to mycobacterial antigens; because of its spontaneous resolution without treatment, TPE is usually considered as a good model for studying the mechanisms of the protective immune response at the sites of *M.tb* infection [3, 4]. T cells and their effector cytokines are critical in the control of *M.tb* infection [5–7]; moreover, emerging evidence indicates that activated CD8^+^ T cells are enriched in TB lung and TPE, and CD8^+^ T cells play important roles in protecting against *M.tb* via releasing cytokines or cytotoxic molecules [8, 9].

Our previous studies found that CD69^+^CD8^+^ T cells were the major source of *M.tb* antigen-specific CD8^+^ T cells in TPE. Following stimulation with *M.tb*-specific peptides, CD69^+^CD8^+^ T cells, but not CD69^−^CD8^+^ T cells, produced high levels of CD107a/b and cytokines [10]. CD69 is well known as an important surface marker expressed on lymphocytes at the early stage [11] and is also considered as an excellent marker of tissue-resident memory T cells (T_RM_ cells) in nonlymphoid tissues [12]. In concert with CD69, integrin *α*_E_ (CD103) is another important marker for especially identifying T_RM_ cells in epithelial tissues. However, lots of studies indicate that T cell recruitment and long-term retention to peripheral tissues are not strictly unaffected by CD103 expression [13, 14].

Unlike traditional effector memory T cells (T_EM_) and central memory T cells (T_CM_), T_RM_ cells persist in epithelial barrier tissues and special organs without recirculating in blood [13, 14]. T_RM_ cells are well known to be the predominant protective cells against viral infection and cancer and also contribute to promoting autoimmunity and allergy diseases [15, 16]. However, the phenotypical characteristics and functions of memory CD103^+^ or CD69^+^CD8^+^ T cells after bacterial infection in humans need more understandings.

Here, we analyze the component of CD103^+^ lymphocytes in TPE and identify three distinct subsets of CD8^+^ T_RM_-like cells in TPE exhibiting phenotypical and functional heterogeneity. We observed that the frequencies of CD103 and CD69 expression on lymphocytes were extremely increased in PFMCs compared with those in peripheral blood mononuclear cells (PBMCs) from patients with TB and healthy donors, and the predominant subset of CD103^+^ lymphocytes in PFMCs was CD8^+^ T cells. Phenotypical analysis indicated that CD103^+^CD69^−^CD8^+^ T cells, but not CD103^+^CD69^+^ or CD103^−^CD69^+^ T cells, were more activated memory T cells. Importantly, CD103^−^CD69^+^ memory CD8^+^ T cells produced more IFN-*γ* after BCG treatment, while CD103^+^CD69^−^ memory CD8^+^ T cells expressed higher cytotoxic and inhibitory molecules. BCG did not upregulate CD103 expression on CD8^+^ T cells, while exogenous TGF-*β* induced CD103 expression on CD8^+^ T cells. Our research appears the immunological characteristics of distinct CD8^+^ T_RM_-like subsets in TPE.

## 2. Materials and Methods

### 2.1. Study Participants

32 patients with TPE (17 males and 15 females, 31-73 years old) were recruited from the Chest Hospital of Guangzhou, China. The diagnosis of pleural effusion (PF) from TB etiology was based on previously described [17]. 17 patients with lung TB (10 males and 7 females, 25-63 years old) were recruited from the Chest Hospital of Guangzhou, 15 healthy volunteers (7 males and 8 females, 19-35 years old) were recruited from Zhongshan School of Medicine, Sun Yat-sen University, and heparinized blood samples were collected from each donor. All PF samples were obtained before the initiation of chemotherapy and antituberculous or steroid therapy at the time of the study, and individuals that had been diagnosed with HIV, hepatitis B virus (HBV), and hepatitis C virus (HCV) infection or with a history of autoimmune diseases were excluded from the study. The patients and donors gave written consent, and the study was approved by the Medical School Review Board at Zhongshan School of Medicine, Sun Yat-sen University.

### 2.2. Antibodies and Reagents

The following monoclonal antibodies were used for phenotypes and intracellular cytokine analyses: PE-CF594 anti-CD3 (UCHT1), APC-Cy7 anti-CD4 (RPA-T4), phycoerythrin (PE) anti-CD69 (FN50), PE anti-CXCR3 (CXCR3-173), PE anti-CD15 (HI98), PE-Cy7 anti-CD69 (FN50), PE-Cy7 anti-CCR4 (1G1), PE-Cy7 anti-CD103 (Ber-ACT8), PE-Cy7 anti-CCR7 (3D12), PE-Cy7 anti-CD19 (HIB19), PE-Cy7 anti-CD56 (B159), PE-Cy7 anti-CD14 (M5E2), Percp-Cy5.5 anti-CD3 (UCHT1), Percp-Cy5.5 anti-CD8 (RPA-T8), Percp-Cy5.5 anti-CD45RO (UCHL1), allophycocyanin (APC) anti-CD8 (RPA-T8), APC anti-CD27 (M-T271), APC anti-CCR6 (11A9), APC anti-CD62L (SK11), APC anti-IFN-*γ* (B27), APC CTLA-4 (BN13), APC anti-*γδ*TCR (B1), APC CD16 (3G8), APC-H7 anti-CD8 (RPA-T8), Alexa Fluor® 647 anti-Granzyme B (GB11), Alexa Fluor® 488 anti-CXCR5 (RF8B2), Alexa Fluor® 700 anti-CD45RO (UCHL1), fluorescein isothiocyanate (FITC) anti-CD103 (Ber-ACT8), FITC anti-CD127 (HIL-7R-M21), FITC anti-CD25 (M-A251), and BV510 anti-CD4 (OKT4) were purchased from BD Biosciences (Franklin Lakes, NJ, USA); APC anti-FoxP3 (236A/E7) and FITC anti-CXCR3 (CXCR3-173) were purchased from eBioscience (San Diego, CA, USA); PE anti-TCRv*α*24 (6B11) was purchased from Beckman Coulter (Brea, CA, USA). LIVE/DEAD fixable violet dead cell stain kit (405 nm excitation) was purchased from Thermo Fisher Scientific (Waltham, MA, USA). Purified anti-CD3 and anti-CD28 monoclonal antibodies (mAbs) were purchased from BD Biosciences. The antibodies to IFN-*γ*, IFN-*α*, TGF-*β*, and TNF-*α* were purchased from PeproTech (Rocky Hill, NJ, USA). Phorbol myristate acetate (PMA), ionomycin, and Brefeldin A (BFA) were purchased from Sigma-Aldrich (St. Louis, MO, USA).

### 2.3. Preparation of PFMCs and PBMCs

The isolation of PFMCs has been described as the previous study [10]. Briefly, erythrocytes were lysed by ammonium chloride solution and resuspended in complete RPMI1640 medium (Gibco, Grand Island, NY, USA) supplemented with 10% fetal calf serum (Sijiqing, Hangzhou, China), 100 U/mL penicillin, 100 *μ*g/mL streptomycin, 50 *μ*M 2-mercaptoethanol, and 2 mM L-glutamine (all from Gibco). PBMCs from heparinized blood were isolated by Ficoll–Hypaque (Haoyang Biological Manufacture, Tianjin, China) gradient centrifugation within 24 hours of blood drawing and suspended in complete RPMI1640 medium.

### 2.4. Cell Culture Conditions

The cells were stimulated with or without immobilized anti-CD3 (1 *μ*g/mL) plus anti-CD28 (1 *μ*g/mL) mAbs (also named as neutral condition) in the presence or absence of cytokines for 0 to 72 hours. For intercellular staining, the cells were stimulated with BCG plus anti-CD28 for 4 hours, and 10 *μ*g/mL BFA was added for additional 8 hours.

### 2.5. Cell Surface and Intracellular Staining

Flow cytometry was operated as described [18]. Briefly, cells were washed twice with PBS buffer containing 0.1% BSA and 0.05% sodium azide. For surface staining, cells were incubated with, respectively, monoclonal antibodies at 4°C in the dark for 30 min. For intracellular staining, cells were fixed in 4% paraformaldehyde, followed by permeabilization, and stained for the intracellular cytokines in PBS buffer containing 0.1% saponin. For FoxP3 staining, 1 mL of 1x fixation and permeabilization solution was added to the cells, incubated for 30~60 minutes at room temperature (purchased from eBioscience), the cells were washed and resuspended in 100 *μ*L of 1x wash buffer, and APC-labelled anti-FoxP3 was added and incubated for 30~60 minutes at 4°C in the dark. All cells were washed twice and fixed in 0.4% paraformaldehyde before the acquisition and collected by BD FACSCalibur and Aria II (Becton Dickinson, San Jose, CA) and analyzed using FlowJo software (Treestar, San Carlos, CA).

### 2.6. Statistical Analysis

The paired Student *t*-test was used to compare the expression of CD103 and CD69 in different sample groups, the expression of phenotypical and functional molecules between distinct lymphocytic subsets, and the difference between two dependent groups (e.g., the cells stimulated by distinct cytokines vs. those only stimulated by anti-CD3 plus anti-CD28). Spearman's correlation was used to analyze the relationship between Granzyme B^+^CD8^+^ T cells and CD103^+^CD8^+^ T cells. All statistical analyses were performed using GraphPad Prism software.

## 3. Results

### 3.1. Higher Expression of CD103 and CD69 on Lymphocytes from PFMCs Compared with PBMCs

**Figure 1:**
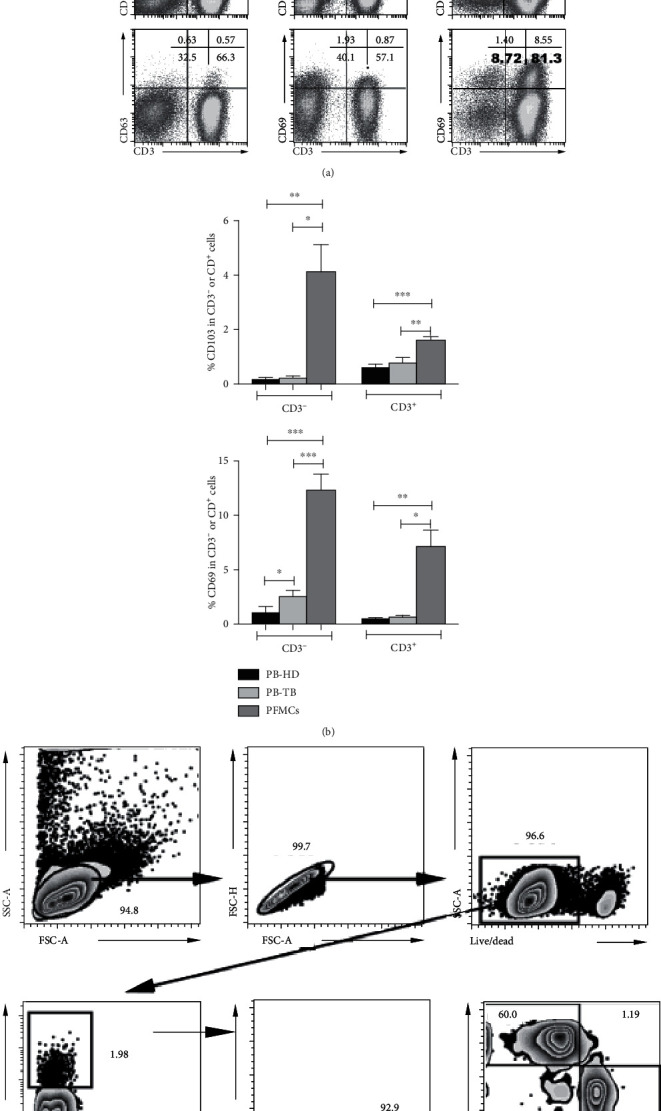
Higher expression of CD103 and CD69 on lymphocytes from PFMCs compared with PBMCs. Comparisons of CD103 and CD69 expression on T cells and non-T cells among PFMCs (PFMCs; *n* = 20) and PBMCs from healthy donors (PB-HD; *n* = 15) and patients with TB (PB-TB; *n* = 17) were analyzed; the (a) representative analysis strategies and dot plots and (b) statistical data were shown. (c) The representative analysis strategies of CD103^+^ lymphocyte subsets were shown, and (d) statistical data showed the frequencies of CD4^+^ and CD8^+^ T in CD103^+^ T cells. ^∗^*P* < 0.05, ^∗∗^*P* < 0.01, and ^∗∗∗^*P* < 0.005.

We have previously found that CD69 was highly expressed on CD4^+^ T cells and CD8^+^ T cells from TPE [10, 19]. In the current study, we observed that both CD103 and CD69 expressions on resting T and non-T cells from PFMCs were significantly higher than those from patients' and healthy donors' PBMCs (Figures 1(a) and 1(b)). Consistent with previous studies [13, 14], CD103^+^CD3^+^ T cells were the major subset of CD103-expressing lymphocytes in PFMCs, and CD103^+^CD8^+^ T cells were the predominant CD103^+^ T cells (Figures 1(c) and 1(d)). Collectively, higher percentages of CD103 and CD69 expression on lymphocytes are observed in PFMCs, and CD103^+^CD8^+^ T cells are the main subset of CD103-expressing cells in PFMCs.

### 3.2. Phenotypical Characteristics of Distinct CD8^+^ T Cell Subsets in PFMCs

**Figure 2:**
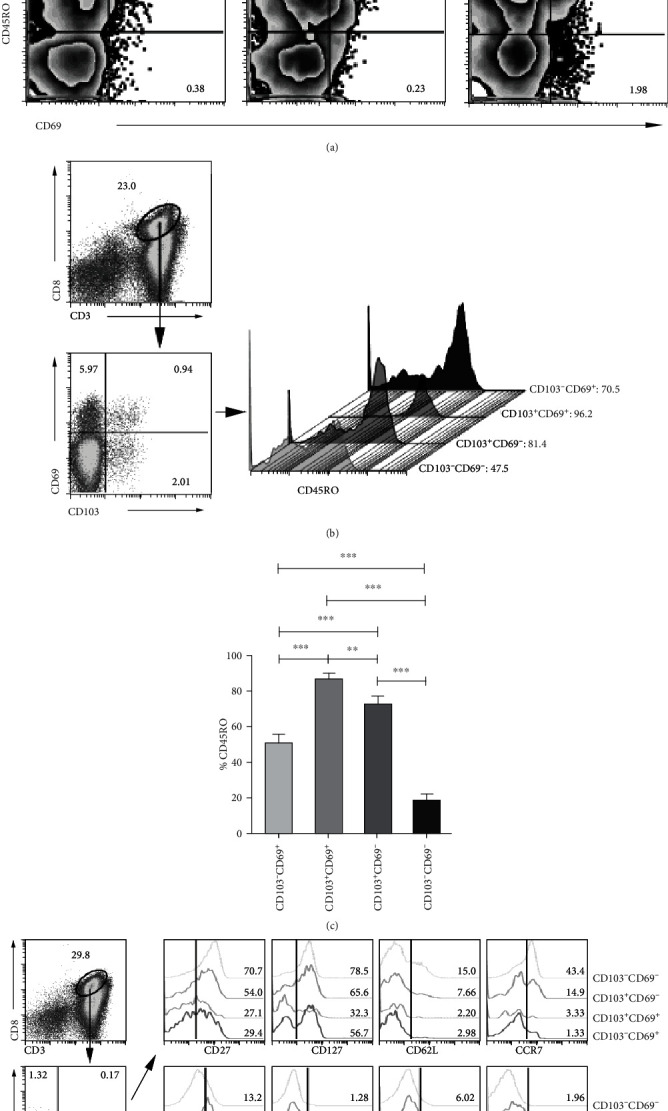
Analysis of surface markers on distinct CD8^+^ T cell subsets. Coexpression of CD45RO and CD103 or CD45RO and CD69 in distinct samples, the representative dot plots were shown (a). The expression of (b, c) CD45RO and (d, e) other surface markers on distinct subsets distinguished based on CD103 and CD69 expression in resting PFMCs was analyzed; the representative dot plots and histogram graphs and statistical data were shown (*n* = 8); ^∗^*P* < 0.05, ^∗∗^*P* < 0.01, and ^∗∗∗^*P* < 0.005.

Next, we detected the phenotypical characteristics of distinct CD8^+^ T subsets. We found that most of CD103-expressing or CD69-expressing CD8^+^ T cells coexpressed CD45RO (Figure 2(a)). Based on CD103 and CD69 expression, CD8^+^ T cells in PFMCs were classified into four subsets, including CD103^−^CD69^+^, CD103^+^CD69^+^, CD103^+^CD69^−^, and CD103^−^CD69^+^. CD103^+^CD69^+^CD8^+^ T cells expressed the highest CD45RO followed by CD103^+^CD69^−^, CD103^−^CD69^+^, and CD103^−^CD69^−^CD8^+^ T cells (Figures 2(b) and 2(c)). In addition, other phenotypical analysis showed that CD103^+^CD69^−^ cells expressed higher levels of CD27, CD127, CD62L, CCR7, CXCR5, and CCR4, while CD103^+^CD69^−^ cells expressed higher levels of CXCR5, CCR4, and CCR6. Compared with CD103^−^CD69^+^ cells, CD127 and CCR7 expression on CD103^+^CD69^+^ cells was lower (Figures 2(d) and 2(e)). Taken together, four CD8^+^ T subsets from PFMCs exhibit distinct phenotypical characteristics, and CD103^+^CD69^+^, CD103^+^CD69^−^, and CD103^−^CD69^+^CD8^+^ T cells exhibit memory phenotype.

### 3.3. Higher Frequencies of CD103^+^ or CD69^+^ Subsets in Memory CD8^+^ T Cells from PFMCs Compared with PBMCs

**Figure 3:**
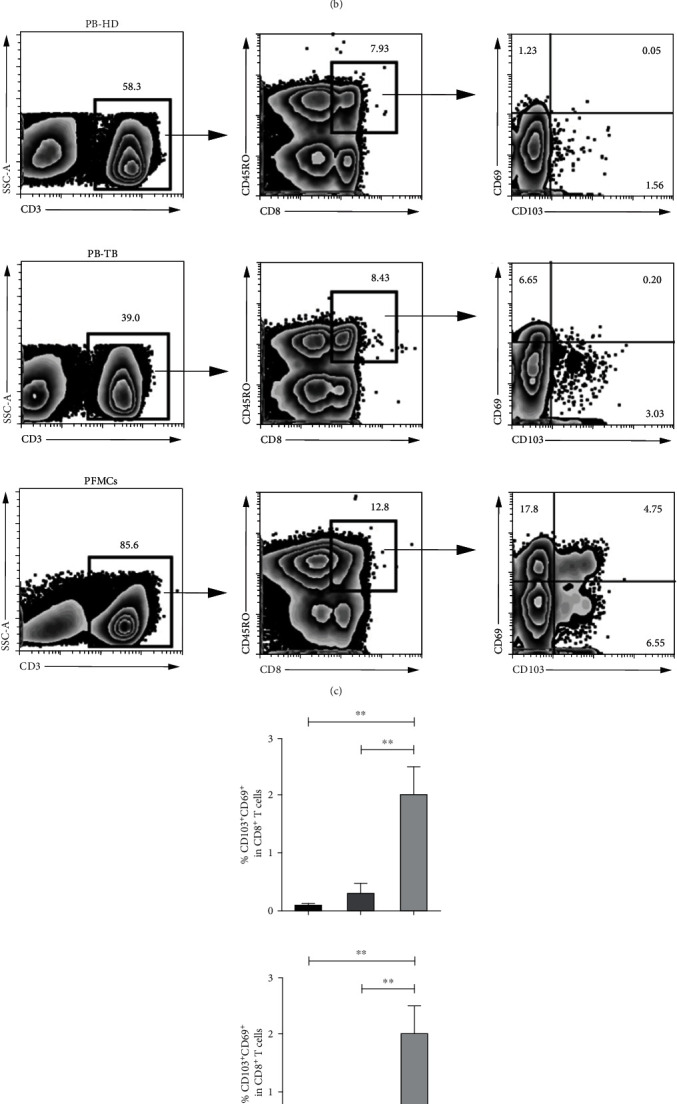
Higher frequencies of CD103^+^ or CD69^+^ subsets in memory CD8^+^ T cells from PFMCs compared with PBMCs. Gated on CD45RO^+^CD8^+^ T cells, the frequencies of CD69^−^CD103^−^, CD69^+^CD103^−^, CD69^+^CD103^+^, and CD69^−^CD103^+^ cells in PFMCs were detected; the (a) representative dot plots and (b) statistical data were shown; the frequencies of CD69^−^CD103^−^, CD69^+^CD103^−^, CD69^+^CD103^+^, and CD69^−^CD103^+^ cells in different samples were compared; the (c) representative dot plots and (d) statistical data were shown (*n* = 8); ^∗^*P* < 0.05 and ^∗∗^*P* < 0.01.

To further understand the characteristics of CD8^+^ memory T cells in PFMCs, we firstly gated on CD45RO^+^CD8^+^ T cells and then divided those memory CD8^+^ T cells into four subsets based on CD103 and CD69 expression. We observed that CD69^−^CD103^−^CD8^+^ T cells were the major subset of memory CD8^+^ T cells followed by CD103^−^CD69^+^, CD103^+^CD69^+^, and CD103^+^CD69^−^ (Figures 3(a) and 3(b)). Furthermore, statistical results indicated that CD103^−^CD69^+^, CD103^+^CD69^+^, and CD103^+^CD69^−^ T cells were extremely increased in memory CD8^+^ T cells from PFMCs without any stimulation in *vitro* (Figures 3(c) and 3(d)). Our results suggest that resting memory CD8^+^ T cells from PFMCs exhibit the phenotype of T_RM_ cells highly expressing CD103 and CD69. However, CD69 is well known as an early active marker; more experiments are needed to determine whether CD69 can be defined as a key marker of T_RM_ cells in PFMCs.

### 3.4. Functional Properties of Different T_RM_-Like CD8^+^ Subsets in PFMCs

**Figure 4:**
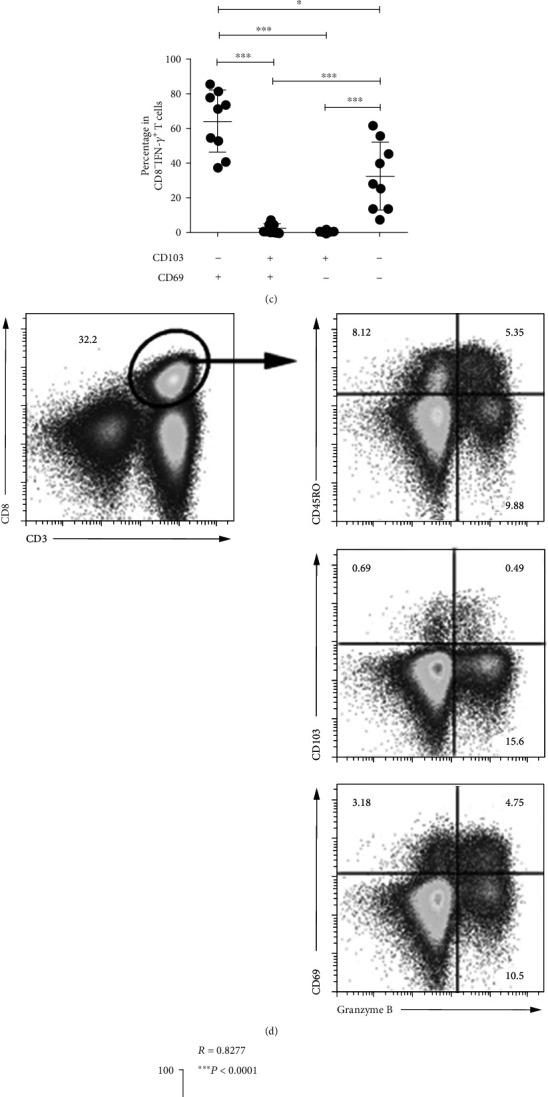
Heterogeneous Granzyme B expression by distinct CD8^+^ T_RM_-like cells from resting PFMCs. PFMCs were stimulated with BCG plus anti-CD28 for 12 hours in the presence of BFA, the coexpression of IFN-*γ*, CD69, and CD103 was detected, and the representative dot plots were shown (a) (*n* = 6). After stimulation with BCG plus anti-CD28, the frequencies of CD69^−^CD103^−^, CD69^+^CD103^−^, CD69^+^CD103^+^, and CD69^−^CD103^+^ cells in IFN-*γ*^+^CD8^+^ T cells were detected; the (b) representative dot plots and (c) statistical data were shown (*n* = 9). The coexpression of Granzyme B and CD45RO, CD103, or CD69 was observed; the (d) representative dot plots were shown, and the correlations between Granzyme B and CD45RO, CD103, or CD69 in resting PFMCs were analyzed (*n* = 27) (e). The expression of Granzyme B by different CD45RO^+^CD8^+^ T subsets distinguished based on CD103 and CD69 expression was analyzed; the (f) representative dot plots and (f) histogram graphs and (g) statistical data were shown (*n* = 5). ^∗^*P* < 0.05, ^∗∗^*P* < 0.01, and ^∗∗∗^*P* < 0.005.

We have previously demonstrated that CD69^+^CD4^+^ and CD69^+^CD8^+^ T cells expressing high IFN-*γ* were the predominant subsets of tuberculous antigen-specific T cells in TPE [10, 19]. Lots of previous studies have confirmed that short-term stimulation of T cells in the presence of secretion inhibitory BFA could block the transport of newly synthesized CD69 molecules to the cell surface [20, 21]. Then, to understand the functions of CD103^+^ T cells, PFMCs were stimulated with BCG for 12 hours in the presence of BFA; the coexpression of CD103 and IFN-*γ* or CD69 and IFN-*γ* was analyzed. Our data showed that most of antigen-specific IFN-*γ*^+^CD8^+^ T cells coexpressed CD69 but not CD103 (Figures 4(a)–4(c)). Mature CD8^+^ T cells play important cytolysis to kill the intracellular microbes infected cells by delivering cytotoxic granule protein [22, 23]; we further analyzed cytotoxic granule protein expression by distinct resting memory CD8^+^ T subsets. The coexpression analysis indicated that some of Granzyme B^+^CD8^+^ T cells were CD45RO^+^, CD103^+^, and CD69^+^ (Figure 4(d)), and the expression of Granzyme B was positively correlated with the CD45RO, CD69, and CD103 expression on resting CD8^+^ T cells (Figure 4(e)). After that, we analyzed Granzyme B expression on distinct memory CD8^+^ T subsets. As expected, CD103^−^CD69^+^ and CD103^+^CD69^+^ memory CD8^+^ T cells expressed higher Granzyme B than CD103^+^CD69^−^ and CD103^−^CD69^−^ memory CD8^+^ T cells did (Figures 4(f) and 4(g)).

**Figure 5:**
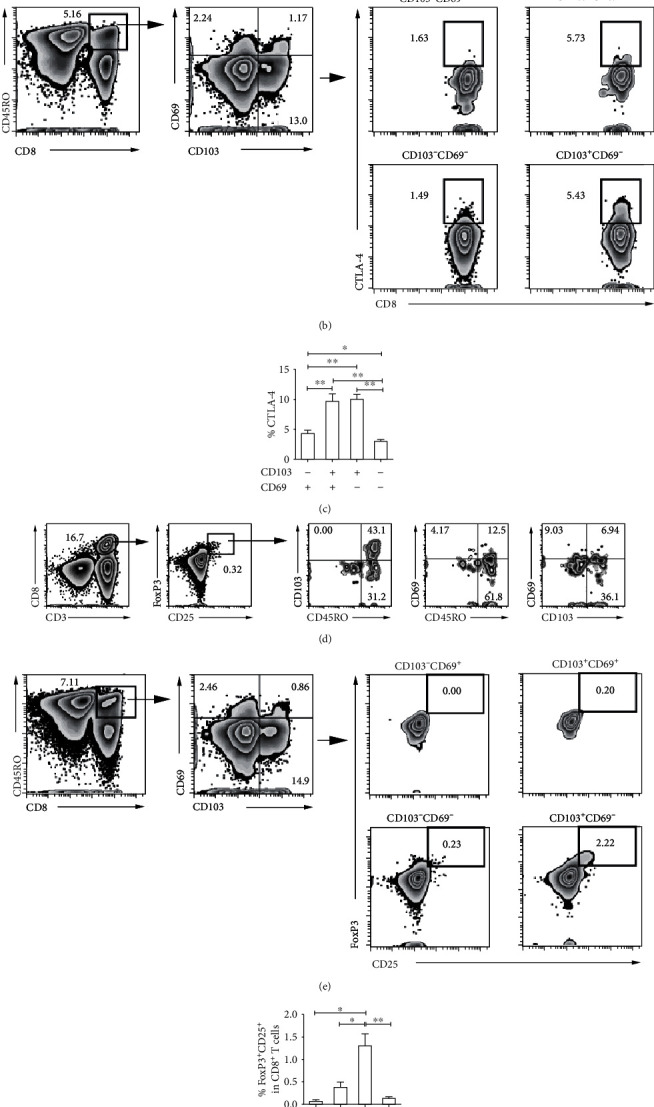
Inhibitory characteristics of CD103^+^CD8^+^ T cells. (a) The phenotype of CTLA-4^+^CD8^+^ T cells from PFMCs, the expression of CTLA-4 on different CD45RO^+^CD8^+^ T subsets distinguished based on CD103 and CD69 was detected, and the (b) representative dot plots and (c) statistical data were shown. (d) The phenotype of FoxP3^+^CD25^+^CD8^+^ T cells from PFMCs, the coexpression of FoxP3 and CD25 in different CD45RO^+^CD8^+^ T subsets distinguished based on CD103 and CD69, and the (e) representative dot plots and (f) statistical data were shown (*n* = 5); ^∗^*P* < 0.05 and ^∗∗^*P* < 0.01.

Overall, to clear the functions of CD103^+^CD69^−^ memory CD8^+^ T subset in PFMCs, we analyzed inhibitory molecule CTLA-4 expression. We observed that some of CTLA-4^+^CD8^+^ T cells coexpressed CD45RO, CD103, and CD69 (Figure 5(a)). Among distinct memory CD8^+^ T subsets, CD103^+^CD69^+^ and CD103^+^CD69^−^CD8^+^ T cells expressed higher CTLA-4 than CD103^−^CD69^+^ and CD103^−^CD69^−^CD8^+^ T cells did (Figures 5(b) and 5(c)). In addition, we also uncovered that some of CD8^+^ T cells from PFMCs expressed FoxP3 and CD25, and most of FoxP3^+^CD25^+^CD8^+^ T cells were CD45RO^+^, half were CD103^+^, and less than 20% were CD69^+^ (Figure 5(d)). The percentages of FoxP3^+^CD25^+^ cells in CD103^+^CD69^−^CD8^+^ T cells were the highest among the four memory CD8^+^ T subsets (Figures 5(e) and 5(f)). Collectively, CD103^−^CD69^+^CD8^+^ T cells in PFMCs might respond to *M.tb* by producing cytokines and cytotoxic granule protein Granzyme B, while CD103^+^CD69^−^CD8^+^ T cells possibly exhibit inhibitory functions via highly expressing CTLA-4, FoxP3, and CD25.

### 3.5. Upregulation of CD103 and CD69 Expression on CD8^+^ T Cells by TGF-*β* and IFN-*α*

**Figure 6:**
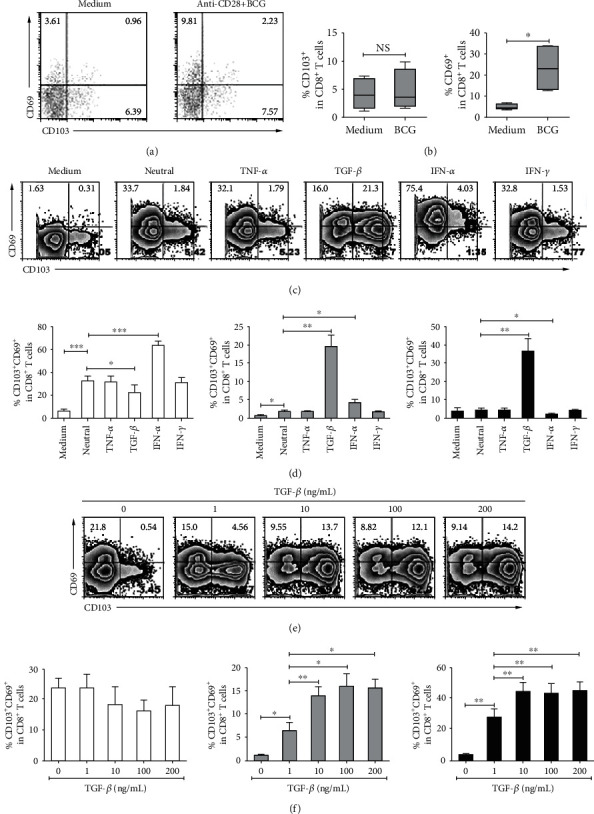
Upregulation of CD103 and CD69 on CD8^+^ T cells by TGF-*β* and IFN-*α*. PFMCs were stimulated with or without anti-CD28 in the presence or absence of BCG for 48 hours, and then, CD69 and CD103 expression on CD8^+^ T cells were detected; the (a) representative dot plots and (b) statistical data were shown (*n* = 8). PFMCs were stimulated with anti-CD3 plus anti-CD28 (neutral condition) in the presence or absence of TNF-*α*, TGF-*β*, IFN-*α*, and IFN-*γ*, the frequencies of CD69^+^CD103^−^, CD69^+^CD103^+^, and CD69^−^CD103^+^ cells were analyzed, and the (c) representative dot plots and (d) statistical data were shown (*n* = 7). PFMCs were stimulated with neutral condition in the presence or absence of different doses of TGF-*β*, the frequencies of CD69^+^CD103^−^, CD69^+^CD103^+^, and CD69^−^CD103^+^ cells were analyzed, and the (e) representative dot plots and (f) statistical data were shown (*n* = 5). ^∗^*P* < 0.05, ^∗∗^*P* < 0.01, and ^∗∗∗^*P* < 0.005.

To further explore the generation of CD103 and CD69 on CD8^+^ T cells, we stimulated PFMCs under different culture conditions. We observed that CD69 expression on CD8^+^ T cells was extremely increased after stimulation with BCG plus anti-CD28 in the absence of BFA; however, CD103 expression on CD8^+^ T cells was not upregulated (Figures 6(a) and 6(b)). TGF-*β* is well considered to driving CD103 expression in skin and other tissues [24, 25]. We detected the kinetic expression of CD103 and CD69 on CD8^+^ T cells from PFMCs under the condition of anti-CD3 plus anti-CD28 in the presence or absence of different recombinational cytokines, including IFN-*γ*, IFN-*α*, TGF-*β*, and TNF-*α*. TCR signals (only anti-CD3 plus anti-CD28) could induce CD69 expression but not CD103. In line with previous reports [24, 25], exogenous TGF-*β* extremely enhanced CD103 expression but not CD69, while IFN-*α* increased CD69 expression but not CD103 (Figures 6(c) and 6(d)). TCR signal with or without IFN-*α* did not increase CD103 expression, while TCR signal extremely activated CD103^+^CD8^+^ T cells to express CD69. Furthermore, TGF-*β* upregulated CD103 expression on CD8^+^ T cells and also increased the generation of CD69^+^CD103^+^CD8^+^ T cells in a dose-dependent manner (Figures 6(e) and 6(f)).

## 4. Discussion

T_RM_ cells are recently described as nonrecruiting memory T cells that persist in peripheral tissues and play critical roles in the pathology of virus infection and cancer [13, 16]. Consistent with our published studies, we confirmed that T cells from TPE significantly expressed higher levels of CD69 than those from PBMCs of patients with TB and matched healthy donors did [10, 19]. Moreover, a similar increase of CD103 expression on PFMCs compared with PBMCs was observed. CD103 is a noteworthy molecule of CD8^+^ T_RM_ cells localizing in the epithelial tissues. In current studies, we found that CD103 was mainly expressed by T cells and other lymphocytes from TPE, including monocytes, NKT, NK, B cells, and *γδ* T cells (data shown in supplementary figure 1). Both CD103^+^CD8^+^ and CD69^+^CD8^+^ T cells were CD45RO^+^ which indicated that CD103^+^CD8^+^ and CD69^+^CD8^+^ T cells were memory T cells; however, most of CD45RO^+^CD8^+^ memory T cells in TPE were CD103^−^CD69^−^. Positive culture of *M.tb* in a proportion of cases show that TPE may be caused by pleural local *M.tb* infection; huge enrichment of memory CD8^+^ T cells was temporarily recruited into the *M.tb* infectious pleural cavity from peripheral blood or lymphoid nodes, which may be the possible explanation of high CD103^−^CD69^−^ memory CD8^+^ T cells in TPE [26, 27].

CD103 has been reported to coexpress CD69 in many T_RM_ cells especially deriving from epithelial tissues [24]; we found that only part of CD103^+^ memory CD8^+^ T cells from TPE coexpressed CD69. CD69^+^ T cells from TPE have been discussed as effector or effector memory T cells (CD45^+^CD45RA^−^CCR7^−^CD62L^−^CD27^−^) and produce high levels of cytokines after *M.tb*-related antigen stimulation [10, 19]. In our study, the enhanced CD27 and CD127 expression on CD103^+^CD69^+^CD8^+^ T cells compared with CD103^−^CD69^+^ and CD103^−^CD69^+^CD8^+^ T cells was observed. CD27 has been discussed to be required for the generation and long-term maintenance of T cell immunity, and it is a strategy to maintain the clonal diverse CD8^+^ T cell responses of low antigen affinity and reactivity to a wide array of mutable pathogens [28, 29]. CD127 also named IL-7R is a receptor for IL-7, and it is important in directing the differentiation, proliferation, and survival of immune cells [30]. Together, higher CD27 and CD127 expression on CD103^+^CD69^+^CD8^+^ T cells suggests that these cells might be more accessible to be activated and long-term maintenance.

Functionally, our data showed that IFN-*γ*^+^CD8^+^ T cells from TPE were CD103^−^CD69^+^CD8^+^ but not CD103^+^CD69^+^CD8^+^ T cells. Further analysis indicated that CD103 expression on CD8^+^ T cells was obviously correlated with cytotoxic effector molecule Granzyme B expression in TPE, and CD103^+^CD69^+^ memory CD8^+^ T cells expressed the highest frequencies of Granzyme B among the four memory subsets. In addition to discussing the ability of IFN-*γ* and Granzyme B secretion, we also investigated the possible immunosuppressive function of memory CD8^+^ T cell subsets. *M.tb*-specific FoxP3^+^ T cells have been currently discussed to enrich in mice peripheral lymphoid nodes after infection with *M.tb*; those Treg-like cells were activated by expressing high amounts of cell surface CD25, CTLA-4, GITR, CD103, and ICOS, but not producing IFN-*γ* [31]. Upregulation of CD103 expression on CD8^+^ T cells has been reported to be associated with the long-term survival of allograft recipients in a rat liver transplantation model and been used to distinguish CD8^+^ Treg cells from non-Treg cells [32, 33]. In our studies, we observed that both CD103^+^CD69^+^ and CD103^+^CD69^−^ memory CD8^+^ T cells expressed high levels of CTLA-4, which suggested that CD103^+^CD8^+^ T cells might play an essential role in controlling over inflammatory response in the inflammation of pleural cavity. Furthermore, we found CD103^+^CD69^−^, but not CD103^+^CD69^+^ memory CD8^+^ T cells, highly expressed CD25 and FoxP3. Together, we suggested that the enhanced CD103^+^CD69^−^ memory CD8^+^ T cells in TPE might be the major source of CD8^+^ iTreg population. However, more additional studies are needed to confirm the existence of CD8^+^ iTreg cells in TPE and TB, and their phenotypes and functions in regulating possible immunological roles in TPE and TB also require further investigation.

TGF-*β* in some particular tissues is considered as the most important molecule to induce CD103 expression on T cells [23, 24]. We found that exogenous TGF-*β* in a dose-dependent manner promoted CD103 expression on T cells in the absence of *M.tb*-antigen in *vitro*. In our previous study, we demonstrated that TPE inhibited the functions of T cells via immunosuppressive factors in *vitro*, and neutralizing antibodies against TGF-*β* could reverse cytokine production by T cells from PFMCs, suggesting that TGF-*β* in TPF might take part in impairing T cell functions [34]. However, the mechanisms of high TGF-*β* in TPE on how to regulate T_RM_-like cell generation and maintenance are not clear. Moreover, we found that IFN-*α* increased the expression of CD69 on CD103^+^CD8^+^ T cells after stimulation with TCR signal. IFN-*α* plays a key role in innate and adaptive immunity and also provides a directly strong third signal to human CD8^+^ T cells resulting in the regulation of critical genes for their overall activation [35].

## 5. Conclusion

Our study highlights three novel memory CD8^+^ T_RM_-like cell subsets in inflammatory TPE that display heterogeneous phenotypes and functions. CD69^+^ memory CD8^+^ T cells expressed more effector cytokines but not CD103^+^. CD103^+^CD69^+^ memory CD8^+^ T cells compared with the other memory CD8^+^ T subsets expressed the highest Granzyme B and CTLA-4, while CD103^+^CD69^−^ memory CD8^+^ T cells possibly exhibited immunosuppressive potential via expressing CTLA-4 and some regulatory T cell-related molecules. Understanding the phenotypical and functional heterogeneity of distinct CD8^+^ T_RM_-like subsets in TPE will provide new theories to develop therapeutic strategies and vaccines for *M.tb* infection.

## Data Availability

The data that support the findings of this study are available from the corresponding author upon reasonable request.

## Supplementary Materials

Supplementary Materials Supplementary Figure 1: CD103^+^ subset composition in PFMCs with TB. Gated on CD103^+^ cells, the percentages of different subsets were detected, including CD4^+^, CD8^+^, TCR*γδ*^+^CD3^+^ T cells, and NKT cells (TCRv*α*24^+^CD3^+^ or CD56^+^CD3^+^), and non-T cells, such as monocytes (CD14^+^CD3^−^), B cells (CD19^+^CD3^−^), and NK cells (CD56^+^CD3^−^); the (a) representative dot plots and (b) statistical data were shown. Gated on different subsets, the percentages of CD103 expression on different subsets were detected; the (c) representative dot plots and (d) statistical data were shown (*n* = 10).
