# Supplementary material for: Tissue-Resident Memory-Like CD8+ T Cells Exhibit Heterogeneous Characteristics in Tuberculous Pleural Effusion
Source: J Immunol Res. 2021 Apr 22;2021:6643808. doi: 10.1155/2021/6643808 (PMC8084674; doi:10.1155/2021/6643808)
Supplement: Supplementary Materials — Supplementary Figure 1: CD103+ subset composition in PFMCs with TB. Gated on CD103+ cells, the percentages of different subsets were detected, including CD4+, CD8+, TCRγδ+CD3+ T cells, and NKT cells (TCRvα24+CD3+ or CD56+CD3+), and non-T cells, such as monocytes (CD14+CD3−), B cells (CD19+CD3−), and NK cells (CD56+CD3−); the (a) representative dot plots and (b) statistical data were shown. Gated on different subsets, the percentages of CD103 expression on different subsets were detected; the (c) representative dot plots and (d) statistical data were shown (n = 10). [file 6643808.f1.docx]

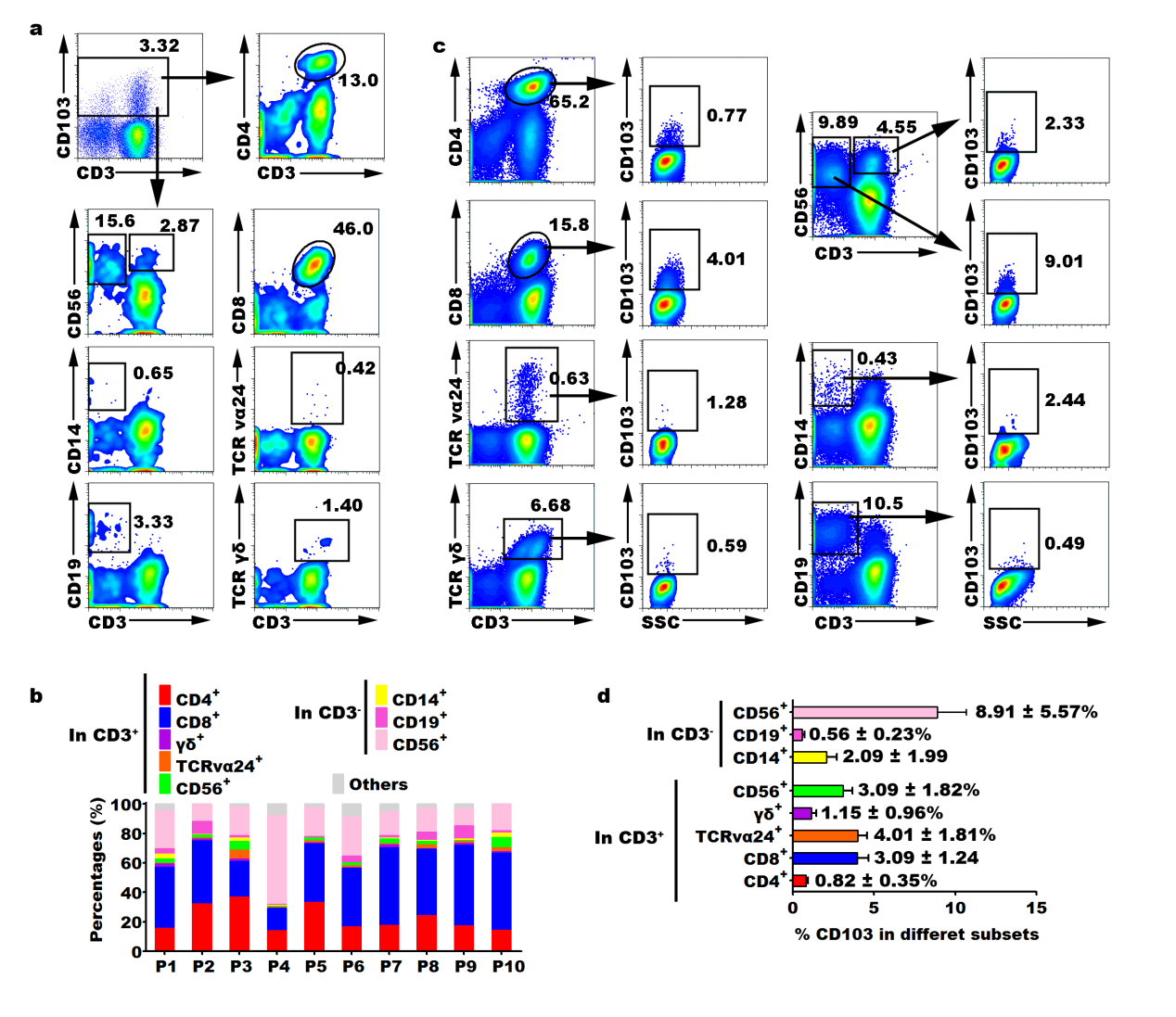


***Supplementary Figure 1: CD103^+^ subset composition in PFMCs with TB.***Gated on CD103^+^ cells, the percentages of different subsets were detected, including CD4^+^, CD8^+^, TCRγδ^+^CD3^+^ T cells and NKT cells (TCRvα24^+^CD3^+^ or CD56^+^CD3^+^), and non-T cells, such as monocytes (CD14^+^CD3^-^), B cells (CD19^+^CD3^-^) and NK cells (CD56^+^CD3^-^),the representative dot plots (**a**) and statistical data (**b**) were shown. Gated on different subsets, the percentages of CD103 expression on different subsets were detected, the representative dot plots (**c**) and statistical data (**d**) were shown, n=10.
